# Editorial: Emerging trends in cardiac and skeletal muscle pharmacotherapy

**DOI:** 10.3389/fphar.2025.1619972

**Published:** 2025-05-15

**Authors:** Young Hoon Son, Ivan Vechetti, Dawn Katrina Coletta

**Affiliations:** ^1^ Biohybrid Systems Group, Coulter Department of Biomedical Engineering, Georgia Institute of Technology and Emory University School of Medicine, Atlanta, GA, United States; ^2^ Department of Nutrition and Health Sciences, University of Nebraska-Lincoln, Lincoln, NE, United States; ^3^ Division of Endocrinology, Department of Medicine, University of Arizona, Tucson, AZ, United States; ^4^ Center for Disparities in Diabetes, Obesity, and Metabolism, University of Arizona, Tucson, AZ, United States; ^5^ Department of Physiology, University of Arizona, Tucson, AZ, United States

**Keywords:** muscle pharmacology, cardiotoxicity, cancer cachexia, translational medicine, therapeutic interventions

Skeletal and cardiac muscles are vital not only for movement but also for maintaining metabolic homeostasis, thermogenesis, and modulating disease progression. Their dysfunction underlies a range of conditions—from cardiovascular and metabolic disorders to cancer cachexia—driving the need for targeted pharmacological solutions. This Research Topic explores the intersection of muscle physiology, molecular pharmacology, and translational medicine, highlighting recent advances in drug development and therapeutic strategies aimed at preserving or improving skeletal and cardiac muscle function.

The Research Topic features five articles that highlight current pharmacological approaches in this field.

One meta-analysis study investigates the protective effects of metformin against doxorubicin-induced cardiotoxicity, a significant clinical limitation of this widely used chemotherapeutic. Using data from animal models, the study reveals dose-dependent effects and identifies underlying mechanisms, particularly oxidative stress reduction and autophagy modulation (Sun et al.).

Another study explores the therapeutic potential of trimetazidine (TMZ) in a rodent model of peripheral artery disease. Through activation of the HIF-1α/VEGF signaling pathway, TMZ promotes angiogenesis and improves perfusion. This research uniquely emphasizes TMZ’s capability to improve ischemic skeletal muscle function—an aspect often overlooked in conventional cardiovascular drug development (Pan et al.).

A review article examines the role of inclisiran, an siRNA-based lipid-lowering agent, across multiple clinical trials. While inclisiran consistently reduces LDL-C levels, most studies focus on biochemical endpoints. This highlights the need for trials that evaluate long-term cardiovascular outcomes and cost-effectiveness, especially in diverse patient populations (Harbi).

In the context of muscle wasting, a novel investigation into Chrysanthemum indicum L. (CI) reveals its efficacy in mitigating cancer cachexia-induced muscle atrophy. CI, and particularly its constituent linarin, improves glucose tolerance and GLUT4 translocation while suppressing proteolytic markers such as MuRF1 and MAFbx, demonstrating comparable or superior effects to celecoxib (Song et al.).

Lastly, a data-driven study explores the role of statins in patients with sepsis-induced myocardial injury. Using the MIMIC-IV database and robust statistical modeling, the authors show that low-dose statin use—particularly simvastatin—is associated with significantly improved short- and long-term survival. These findings may prompt reconsideration of early statin initiation in ICU protocols (Liu et al.).

Together, these contributions expand the translational potential of pharmacological strategies targeting skeletal and cardiac muscle. They embody the core vision of this Research Topic: to bridge molecular discoveries with clinical application, ultimately advancing the development of safe and effective therapies for muscle-related diseases and conditions. We hope this Research Topic inspires continued interdisciplinary collaboration and innovation in the field of muscle pharmacology.

